# Supplementary material for: Correlative microscopy approach for biology using X-ray holography, X-ray scanning diffraction and STED microscopy
Source: Nat Commun. 2018 Sep 7;9:3641. doi: 10.1038/s41467-018-05885-z (PMC6128893; doi:10.1038/s41467-018-05885-z)
Supplement: Supplementary file 1 — Supplementary Information [file 41467_2018_5885_MOESM1_ESM.pdf]

Supplementary Information

Correlative microscopy approach for biology using x-ray holography, x-ray scanning diffraction and STED microscopy

Bernhardt et al.

1 Supplementary Note 1

Implementation of a microscope into the synchrotron instrument. A dedicated STED microscope was designed to meet the special demands of a synchrotron beamline. The optical beam axis was anti-parallel to the x-ray beam, along the horizontal direction. Figure 1 shows photographs of the STED microscope implemented into the GINIX instrument. Supplementary Fig. 1a: The STED microscope consists of a ① computer rack including the excitation and the STED Laser, the avalanche photodiode (APD) as well as the control computer, two ② glass fiber cables with each of about 10 m in length and an ③ optics box including all optical components. The STED beam is focused through a ④ high NA air objective. The sample is glued on a brass pin and then mounted on a ⑤ motorized sample tower. This tower is composed of a hexapod on a long linear translation rail, for translation of the sample over a distance of about 30 cm between STED and x-ray focus along the y-direction. The optics box of the STED microscope and sample tower are installed on a ⑦ granite block to minimize vibrations.

Before recording, a ⑧ safety cap can be placed on top of the sliding carriage. The cap prevents the STED beam to exit the immediate sample environment. The signals from two contact switches at the sides are fed back to the STED microscope interlock system ensuring that no beam can exit the Laser box. In this way, no further connection to the beaprincipal component amline interlock system is required and the microscope can be used as a standalone instrument. Alternatively, the cap can be disposed of, when the beamline interlock system is used. Transmission and reflection illumination is enabled by two white-light LED rings. The positioning of the sample with respect to the (air) objective with a free working distance of 180 micrometers can be monitored by a ⑨ vertical video-microscope. The sample is first inspected in the bright field mode to adjust the sample orientation and to navigate to a particular region of interest. Next, epifluorescence mode is applied in order to coarsely identify the target structure of a particular cell and its fluorescent labeling intensity. Further, the focal plane is coarsely adjusted. Next, a confocal overview scan (step size typically 200 nm) is acquired. An additional confocal scan along the optical beam axis can be used to adequately place the sample in the focal plane of the STED objective. Finally, a combined confocal and STED scan is carried out with a typical stepsize of around 30 nm.
The STED micrograph shown in Fig. 2a (main article) was recorded with a scanning stepsize of $\Delta_{y,z} = 30\,\text{nm}$ and a dwell time of $\tau = 0.29\,\text{ms}$. The maximal average STED-Laser power is 1.25 W. For the scan of Fig. 2a (main article), the depletion power was lowered to 80 %, which equals an output power of 1 W. Supplementary Fig. 1b-d shows selected photographs of the instrument in different working states. Supplementary Fig. 1b: Freeze-dried cardiac tissue cells adhered to a glass cover slip are positioned in the focal plane of the STED objective. The coverslip is sectioned with a red cryomarker for better orientation and navigation on the sample using bright field mode. The luminescent red dot originates from the (highly attenuated) epifluorescence-LED-illumination. Here, this helps to identify the current position of the probing spot on the sample plane. An upgraded setup including a new hexapod sample tower, see Supplementary Fig. 1c, allows to eliminate tilts of the sample substrate, so that the substrate surface can be aligned parallel to the focal plane of the STED microscope. The corresponding alignment procedure is facilitated by a top- and a side-view video camera, which can monitor the approach of the substrate surface to the objective. For holographic recordings, the sample is placed into a defocus plane with respect to the x-ray waveguide, which is positioned on a vertical hexapod (Smaract GmbH, Germany) mounted onto the evacuated KB mirror tank (upper right in the back), see Supplementary Fig. 1d. An on-axis video (OAV) microscope (lower left in the front) helps to re-identify the specific region imaged by STED microscopy before (in addition to pre-calibrated motor positions). A hole drilled into the objective of the OAV allows for simultaneous operation during the x-ray recording.

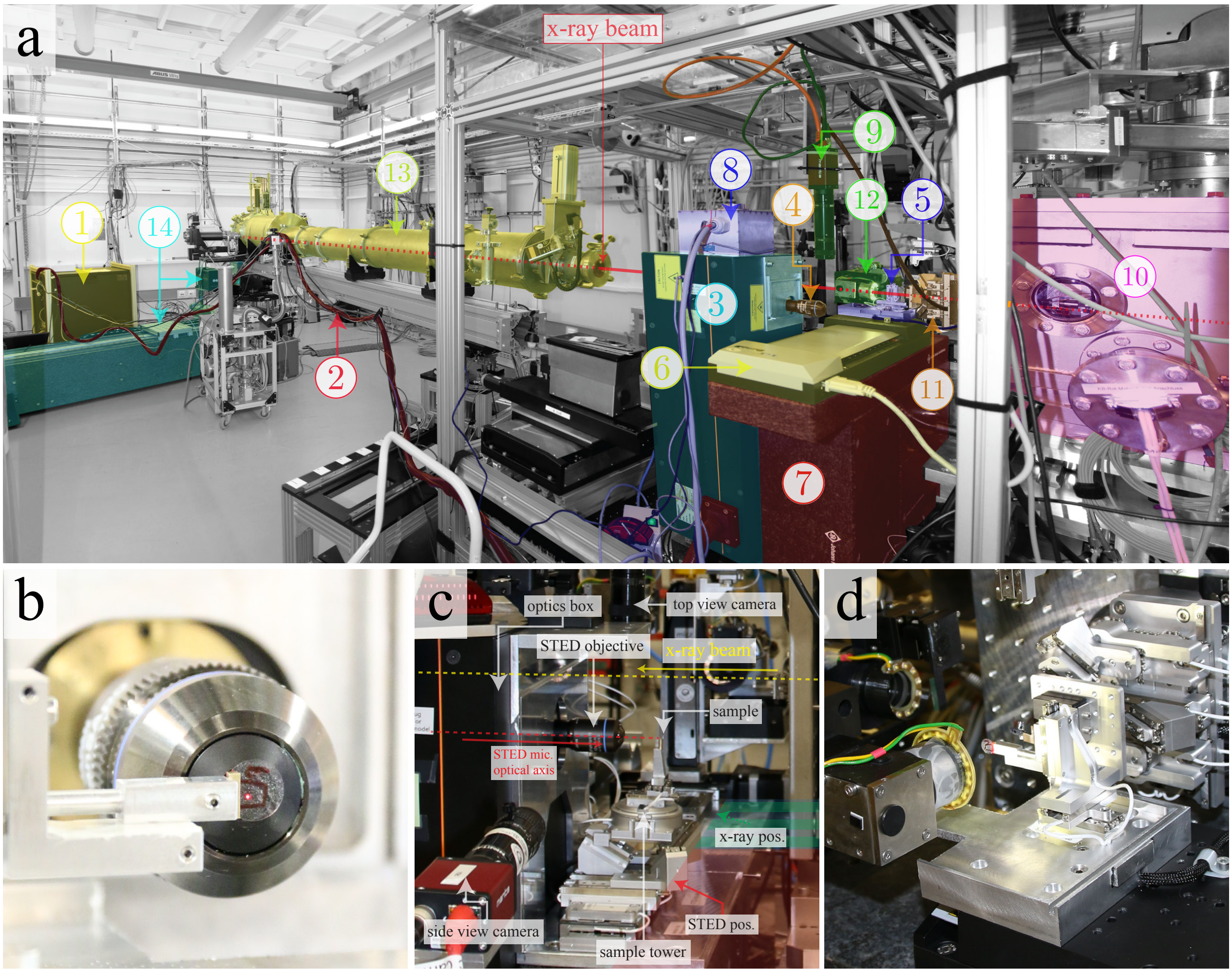

Supplementary Figure 1: Photographs of the combined imaging setup. (*a*) Composite photograph of the STED microscope as incorporated in the Göttingen Instrument for Nano-Imaging with X-Rays `GINIX` at P10-beamline. Colors mark the different major components of the combined setup. (1) STED microscope rack, (2) glass fiber cable, (3) optics box, (4) STED microscope objective, (5) sample & motorized tower, (6) stepmotor for y-translation, (7) granite blocks, (8) safety cap & brightfield illumination, (9) top view video camera, (10) KB-box, (11) waveguide & xyz-mount, (12) on axis video camera, (13) evacuated tube, (14) x-ray detectors & bench. (*b*-*d*) shows photographs of the combined setup in different working states. (*b*) Freeze-dried cardiac tissue cells on a glass cover slip in the STED recording position. The coverslip is sectioned by a cryomarker. The luminescent red dot originates from the epifluorescence-LED inside the optics box of the STED microscope. (*c*) Photograph of the current setup including a hexapod. The hexapod sample tower is mounted on a rail perpendicular to the STED- (red line shown in front) and x-ray beam axis (yellow line shown in the back) in order to be able to overcome the distance of about 30 cm between the STED- and the x-ray recording position. (*d*) Sample in the x-ray recording position inspected by the fully motorized on-axis video microscope (with white luminescent LED-ring).

2 Supplementary Methods

Segmentation algorithm. For segmentation analysis, data of the STED micrograph and the RAAR reconstruction (with maximum field of view) were exported as grayscale images and then processed by the filament sensor, see (1), with the parameters as listed in Supplementary Tab. 1. The filament sensor algorithm can be downloaded from ref. (2).

STED micrograph	
Quality Score	858
Scale	1
Minimal contrast	0
Set contrast manually	✓
black value	0
white value	138
Remove standalone pixels	✓
Laplace filter	✓, 8 neighbor
Factor	2
Gaussian filter	✓
Sigma	1
Directed Faussian filter	✓
Restrict to Area Mask	✓
Sigma	10
Image filter	Gauss first
Binarized method	Directions
Minimum mean value	40
Sigma	2
Minimum standard deviation	2
Curved filamets	✓
Minimum filament length	25
Length of straight pieces	5
Minimum angle difference	10
Tolerance	5 %
Log Scale	✓, Lines as samples
Minimum area	5 %
Minimum filaments	5

RAAR reconstruction	
Quality Score	838
Scale	1
Minimal contrast	30
Set contrast manually	✓
black value	10
white value	171
remove standalone pixels	✓
Laplace filter	✓, 8 neighbor
Factor	4
Gaussian filter	✓
Sigma	3
Directed Faussian filter	✓
Restrict to Area Mask	no
Sigma	10
Image filter	Gauss first
Binarized method	Directions
Minimum mean value	32
Sigma	1.8
Minimum standard deviation	3
Curved filamets	✓
Minimum filament length	30
Length of straight pieces	20
Minimum angle difference	20
Tolerance	5 %
Log Scale	✓, Lines as samples
Minimum area	5 %
Minimum filaments	5

Supplementary Table 1: Parameters for the STED and the RAAR segmentation analysis.

Holographic reconstruction by CTF & RAAR algorithms. X-ray holograms were recorded at 5 m distance, with the cell being placed about 25 mm behind the waveguide. The waveguide consisted of a 80 nm thick C guiding layer, sandwiched between Mo cladding layers in crossed geometry (3). For empty beam recordings, the cell was moved out of the beam laterally. The empty beam divided holograms were treated by frequency filters to compensate artifacts induced by wavefront drift between recordings of the cell and the empty beam. Data was reconstructed by imposing the constraints of pure phase object, as well as a support constraint (for RAAR). The support for the RAAR reconstruction was generated from a CTF reconstruction, see Supplementary Fig. 2. The parameters of both the CTF reconstruction (single step, non-iterative) and the RAAR are listed in Supplementary Tab. 2.

CTF	
Fresnel	0.00047
lim1	0.80
lim2	0.23

RAAR	
Fresnel	0.00047
β_0	0.90
β_m	0.50
β_s	50
iterations	250

Supplementary Table 2: Parameters for CTF and RAAR reconstruction.

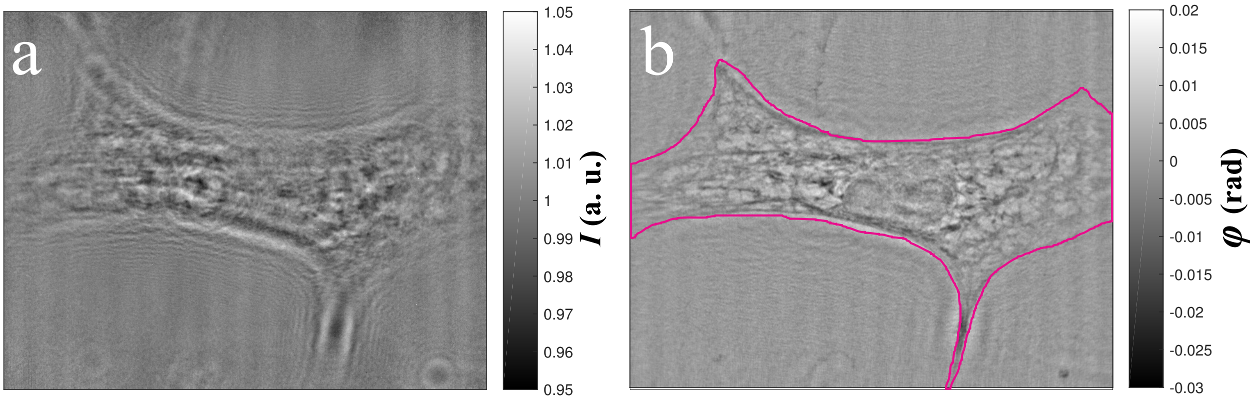

Supplementary Figure 2: Definition of sample support. (*a*) Filtered hologram together with (*b*) CTF reconstructed cell including the support constraint (pink frame) defined on the CTF image and used for the RAAR reconstruction.

Scanning SAXS and principal component analysis. For x-ray dark field analysis of the scanning SAXS measurement, the overall scattering power for each scan position was determined by integration of the photon counts of each pixel on the detector plane, see Fig. 2 (main article) and Supplementary Fig. 3a. Primary beam intensities were excluded by applying a binary mask, setting pixel values around the beam stop to zero, see Supplementary Fig. 3b, black frame. The corresponding dark field map further allows to separate nucleic (a, pink frame), cytoplasmic (a, beige frame) and background regions (a, blue frame). For principal component analysis (PCA), diffraction patterns were processed by a missing data relief function ('healing' function of the nano-diffraction toolbox described in ref. (4) and made available at GitHub ref. (5)), to fill the gaps caused by the beam stop holder (marked in b by △), and the horizontal intermodular gaps of the p300k detector (marked in b by ∗). Moreover, each pattern was corrected by subtracting an average background pattern derived from the blue-framed region of a. Before PCA, a radial binary mask was applied, excluding the primary beam intensities. Supplementary Fig. 3c shows an example of a healed single diffraction pattern including the applied radial binary mask (area framed by two red dashed circles) used for PCA.

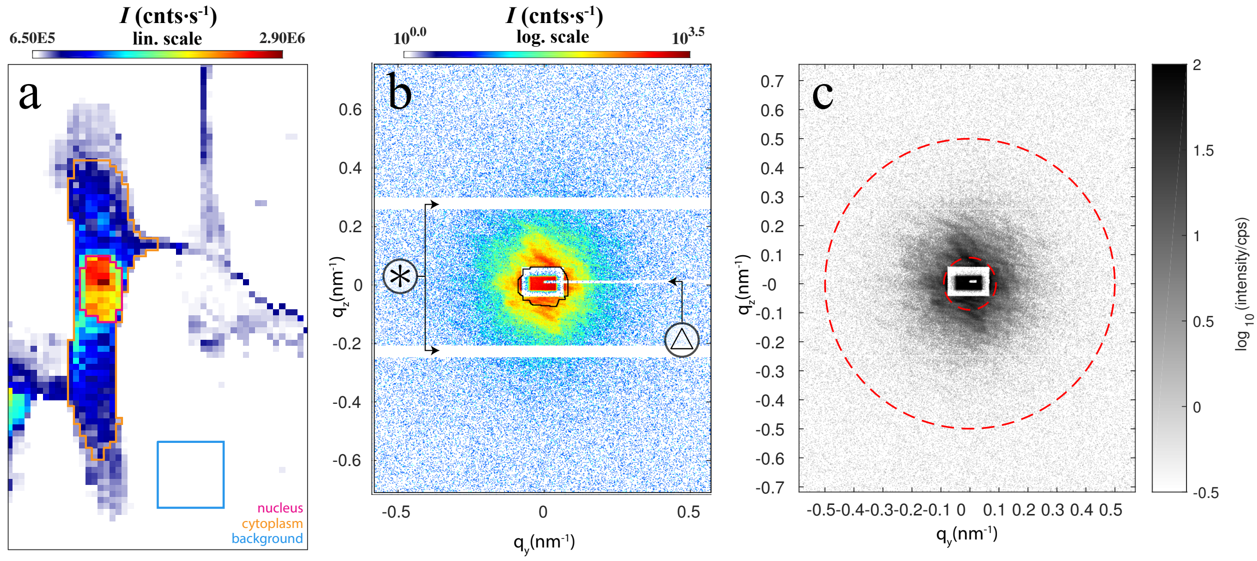

Supplementary Figure 3: Data processing and masks for scanning SAXS analysis. (*a*) X-ray dark field including the STXM-masks used for principal component analysis of Fig. 3c of the main article as well as for the determination of the scattering distribution of Fig. 4 of the main article. The masks section the dataset into a nucleic (pink), a cytoplasmic (beige) and a background compartment (blue). (*b*) Single diffraction pattern as shown in Fig. 4 (inset) of the main article including the used dark field evaluation mask framed in black. △ and ∗ labels mark the detector areas with missing photon counts. The loss of signal can be attributed to the beamstop/beamstopholder as well as to the two intermodular gaps of the detector panel, respectively. (*c*) Example of a healed single diffraction pattern including the applied radial binary mask (interspace between the two red dashed circles) used for PCA.

Numerical rebinning, overlay of data and translational shifts. In order to overlay the three different datasets, pixel sizes of the RAAR reconstructed image as well as of the x-ray dark field were adjusted to the step size of the STED recording using the imresize function of Matlab. Scaling factors are simply the ratio of $p_{\text{eff}}/\Delta_{\text{STED}}$ and $\Delta_{\text{sSAXS}}/\Delta_{\text{STED}}$, respectively, with the effective pixel size p_{eff} of the RAAR reconstruction, and the scanning stepsizes of the STED recording Δ_{STED} as well as of the scanning SAXS recording Δ_{sSAXS}. Translational shifts were determined 'by eye' successively superimposing the STED micrograph, the RAAR reconstructed result and the x-ray dark field from scanning SAXS. Figure 4 shows the final result of the pairwise positioning between the STED micrograph (red) and the RAAR reconstruction (blue) in a, the STED micrograph (red) and the x-ray dark field (green) in b, and the RAAR reconstruction (blue) and the x-ray dark field (green) in c.

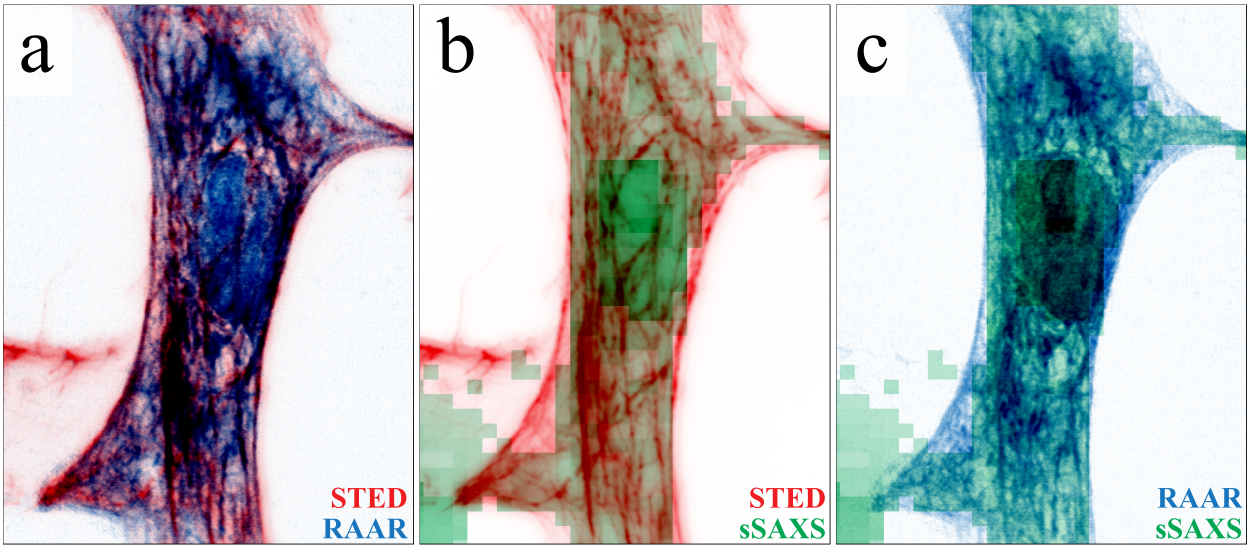

Supplementary Figure 4: Superposition of datasets. (*a*) STED micrograph (red) & RAAR reconstruction (blue). (*b*) STED micrograph (red) & x-ray dark field (green). (*c*) RAAR reconstruction (blue) & x-ray dark field (green).

Supplementary Fig. 5 shows the resulting filaments as found by the filament sensor on the STED micrograph and the RAAR reconstruction as a superposition of datasets. Filaments of the STED micrograph are shown in red, filaments of the RAAR reconstruction are shown in blue and area matches between both datasets are depicted in orange. Inset shows the results of the principal component analysis derived from scanning SAXS as in Fig. 3c of the main article.

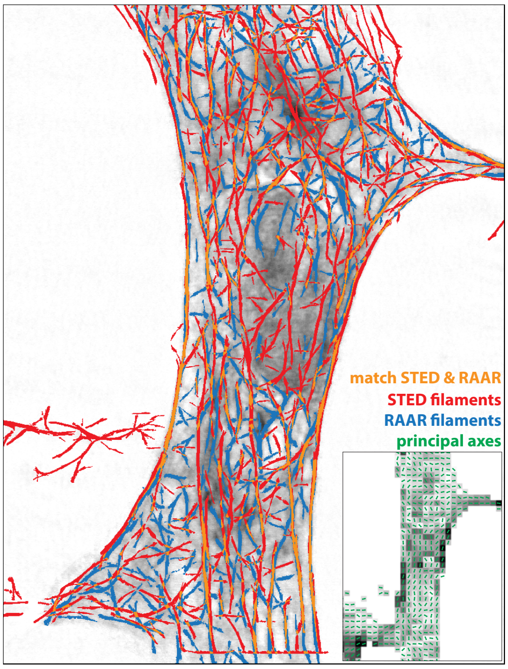

Supplementary Figure 5: Superposition of filaments tracked on the STED micrograph (red), the RAAR reconstruction (blue) and area matches between the two (orange). The ratio of orange pixels to orange & red pixels is about 0.25, indicating that a significant amount of actin filaments visualized by STED microscopy also appear in the holographic reconstruction, presumingly because these filaments are thick enough to stand out in electron density contrast. The inset shows the main elongation axis for each diffraction pattern of the array as found by PCA (green).

Supplementary References

[1] B. Eltzner, C. Wollnik, C. Gottschlich, S. Huckemann, and F. Rehfeldt. The filament sensor for near real-time detection of cytoskeletal fiber structures. *PLoS ONE*, 10(5):e0126346, 2015.

[2] http://filament-sensor.de/.

[3] S. P. Krüger et al. Sub-10 nm beam confinement by x-ray waveguides: design, fabrication and characterization of optical properties. *Journal of Synchrotron Radiation*, 19(2):227–236, 2012.

[4] J.-D. Nicolas, M. Bernhardt, A. Markus, F. Alves, M. Burghammer, and T. Salditt. Scanning x-ray diffraction on cardiac tissue: automatized data analysis and processing. *Journal of Synchrotron Radiation*, 24(6):1163–1172, 2017.

[5] https://irpgoe.github.io/nanodiffraction/.
